# Sale of WHO AWaRe groups antibiotics without a prescription in Pakistan: a simulated client study

**DOI:** 10.1186/s40545-020-00233-3

**Published:** 2020-08-03

**Authors:** Zikria Saleem, Mohamed Azmi Hassali, Brian Godman, Munazzah Fatima, Zeenia Ahmad, Areeba Sajid, Inaam Ur Rehman, Muhammad Umer Nadeem, Zaida Javaid, Madeeha Malik, Azhar Hussain

**Affiliations:** 1 School of Pharmaceutical Sciences, Universiti Sains Malaysia, George Town, Malaysia; 2 The University of Lahore, Lahore, Pakistan; 3 Division of Clinical Pharmacology, Karolinska University Hospital Huddinge, Karolinska Institute, Stockholm, Sweden; 4 Strathclyde Institute of Pharmacy and Biomedical Sciences, Strathclyde University, Glasgow, UK; 5 Health Economics Centre, University of Liverpool Management School, Liverpool, UK; 6 University College of Pharmacy, University of the Punjab, Lahore, Pakistan; 7 Punjab Institute of Cardiology, Lahore, Pakistan; 8 Hamdard Institute of Pharmaceutical Sciences, Hamdard University, Islamabad, Pakistan

**Keywords:** Antibiotics, Sale without prescription, Simulated client, Pakistan

## Abstract

**Introduction:**

Resistant strains of bacteria are rapidly emerging with increasing inappropriate use of antibiotics rendering them less efficacious. Self-purchasing of antibiotics particularly for viral infections is a key driver of inappropriate use, especially in lower- and middle-income countries. There is a particular issue in countries such as Pakistan. Consequently, there is a need to assess current rates of self-purchasing especially for reserve antibiotics to guide future policies.

**Aims:**

Assess the extent of current antibiotic sales without a prescription in urban areas of Pakistan.

**Methodology:**

A multicenter cross-sectional study was conducted in different areas of Punjab, Pakistan using Simulated Client technique. The investigators demanded different predefined antibiotics from WHO AWaRe groups without prescription. Three levels of demand were used to convince the pharmacy staff in order to dispense the antibiotic without a prescription. A data collection form was completed by simulated clients within 15 min of each visit.

**Results:**

Overall 353 pharmacies and medical stores were visited out of which 96.9% pharmacies and medical stores dispensed antibiotics without demanding a prescription (82.7% at demand level 1 and 14.2% at demand level 2), with only 3.1% of pharmacies refusing to dispense antibiotics. The most frequently dispensed antibiotic was ciprofloxacin (22.1%). Surprisingly, even the reserve group antibiotics were also dispensed without a prescription. In only 25.2% visits, pharmacy staff guided patients about the use of antibiotics, and in only 11.0% pharmacists enquired about other medication history.

**Conclusion:**

Currently, antibiotics are easily acquired without a legitimate prescription in Pakistan. There is a need for strict adherence to regulations combined with a multi-dimensional approach to enhance appropriate dispensing of antibiotics and limit any dispensing of WHO restricted antibiotics without a prescription.

## Introduction

Approximately 700,000 people die across the world each year due to drug-resistant infections, with the potential of rising to ten million by 2050 unless actions are taken [1]. The irrational, indiscriminate, and excessive use of antibiotics, including for self-purchasing of antibiotics, is a key driver of increasing antimicrobial resistance (AMR) rates, increasing morbidity, mortality and costs, and is a considerable public health concern [1–8]. As a result, combating resistant bacteria and preventing its transmission has become more challenging, and this will continue in the coming years unless irrational use is reduced [9]. In ten years, from 2000 and 2010, there was 35% increase in the use of antibiotics in which lower and middle-income countries (LMICs), including Russia, Brazil, China, India and South Africa, accounted for 76% of this increase [10]. Due to the marked consumption of antibiotics, resistant strains of bacteria are rapidly emerging rendering them less efficacious [7, 11]. This has resulted in activities globally, regionally and nationally to try and reduce unnecessary prescribing and dispensing of antibiotics [4, 7, 12–17]. Activities also include the World Health Organization (WHO) in 2017 categorizing antibiotics into 3 different groups in order to rationalize their prescribing and dispensing, naming them as Access, Watch and Reserve (AWaRe) antibiotics [18]. The Access group covers first and second line choices for the empiric treatment of the most common infections. The Watch group includes antibiotics with higher resistance potential with their use limited to a lesser number of patient groups. The Reserve group of antibiotics should only be used as a “last resort” treatment option [18, 19].

The major cause of resistance comes from the easy availability of antibiotics with pressure on physicians and pharmacists to prescribe and dispense antibiotics for predominantly self-limiting conditions, aided by poor hygiene and living conditions in a number of LMICs fueling infection rates [7, 20–24]. In a number of countries, antibiotics are largely sold without a prescription in medical stores and community pharmacies adding to AMR rates especially if they are dispensed for viral infections such as coughs and colds [22, 25–32]. There is also poor knowledge among patients and health care professionals on how to properly use antibiotics and the right quantity taken as well as a belief that antibiotics can cure viral diseases such as a cold and influenza [33, 34]. According to a survey conducted by the WHO in Egypt, Sudan and China, around 56% of the population stopped taking antibiotics as soon as they started to feel better [35].

Overall, a lack of diagnostic facilities and poor practices among health care professionals, coupled with public behavior including pressurizing pharmacists to dispense antibiotics for predominantly viral infections, have contributed towards increases in the sale of antibiotics without prescription especially in developing countries [7, 22, 26, 29, 31, 36, 37]. Community pharmacists are also dispensing antimicrobials without prescription in order to nurture the loyalty of customers and to prevent losing them to local competitors [32, 38–40].

We are aware of high rates of self-prescribing of antimicrobials and other medicines in Pakistan often for economic reasons [28, 41–48]. This is despite the Drug Act 1967 of Pakistan stating that antibiotics are not over the counter medications and their availability to the public is illegal without a prescription [49]. In addition, we are aware that variable knowledge of regarding antibiotics and AMR among patients and pharmacists has enhanced self-purchasing in Pakistan thereby increasing resistance rates [50–53], with high resistance rates already evident in Pakistan [50]. Consequently, we sought to add to this knowledge base by assessing current prevalence rates of antibiotic sales without a prescription in urban areas of Pakistan by using the Simulated Client Techniques. In addition, assess the quality of pharmacy services provided to patients in both small- and large-scale pharmacies and medical stores to provide future guidance to the authorities in Pakistan as they develop additional programs to combat AMR as part of the recently developed National Action Plan in Pakistan [17]. Community pharmacists are a key stakeholder with reducing inappropriate dispensing of antibiotics without prescriptions as they are often the first healthcare professionals that patients consult with especially in LMICs where affordability is a key issue with often insufficient financial means to see both a physician and purchase medicines [50, 54–56].

## Methods

A multicenter cross-sectional study in pharmacies and medical stores of different cities of Pakistan was conducted using the Simulated Client technique, which is a well-recognized technique to help accurately assess pharmacist and dispenser activities [56–61]. The study was approved by the Human Ethics Committee for Clinical Research of the Punjab University College of Pharmacy, University of the Punjab, Lahore, Pakistan (HEC/1000/PUCP/1925WP). Pharmacies in Pakistan are currently run under the supervision of registered pharmacist, whereas, medical stores where medicines can also be dispensed are supervised by registered dispensers [49], although this is likely to change. A total of 353 pharmacies and medical stores were selected using non-probability convenience sampling technique. The sample size was calculated using the online sample calculator Raosoft (http://www.raosoft.com/samplesize.html) by keeping 5% margin of error, 95% confidence interval, and 50% response distribution.

**Fig. 1:**
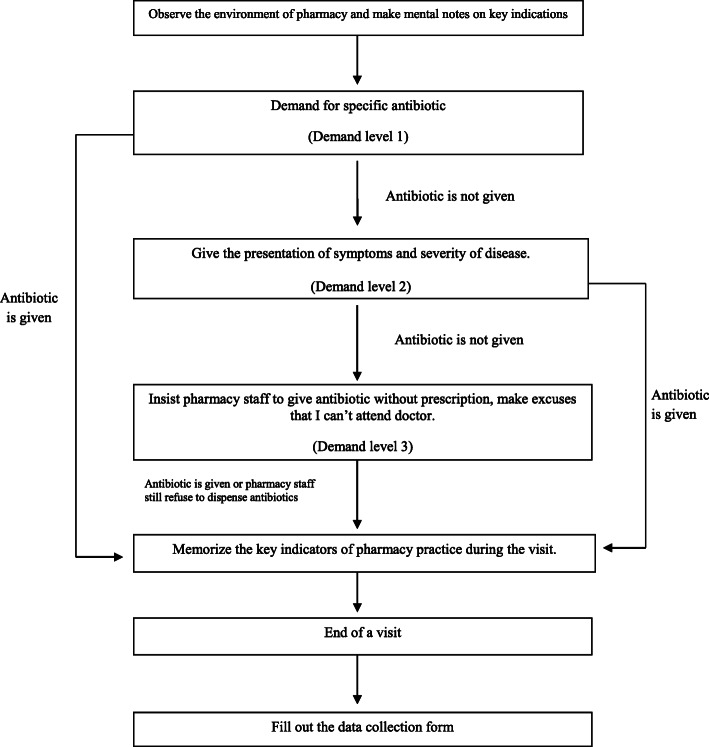
Flowchart of the pre-designed visiting process

Pharmacy undergraduates from the University College of Pharmacy, University of Punjab, were recruited as simulated clients and they underwent training. They were advised to select the pharmacy or medical store in their own area and demand any of the access, watch or reserve (AWaRe) group of antibiotics according to predesigned standardized visiting process (Fig. 1 and Additional file 1). All the essential details regarding the study were provided to the undergraduates in advance in order to ensure a smooth performance at the time of their visit. Investigators approached the staff as normal customers without creating any suspicion. The investigators represented their demands utilizing three direct product request (DPR) levels until an antibiotic was dispensed or denied (Fig. 1). Figure 1) illustrates our general methodology in the form of a flow chart. The first thing our simulated client did was he/she observed the environment of the pharmacy and made mental notes about key indications. Then the client generally asked for an antibiotic from the pharmacy without showing any prescription or sharing any symptoms. We specified this as Demand level 1. If the antibiotic was given to our client, he/she memorized the key indicators of pharmacy practice during the visit and ended the visit (Additional file 1). Then he/she filled out the data collection form. If the antibiotic was not given to our client, the client subsequently gave the presentation of symptoms and severity of disease which could be either an respiratory tract infection or diarrhoea (Additional file 1). We specified this as Demand level 2. Again, if the antibiotic was given on Demand level 2, the client memorized the key indicators of pharmacy practice during the visit and ended the visit just like before and filled out the data collection form. However, if the antibiotic was still not given our client insisted the pharmacy staff dispense an antibiotic without a prescription and made excuses that he can’t attend the doctor. We specified this as Demand level 3, and in this level whether the antibiotic was given to our client or not, he/she just memorized the key indicators of pharmacy practice during the visit and subsequently ended the visit and completed the data collection form. The simulated clients recorded all the information including whether an antibiotic was dispensed or not, the type of antibiotic that was dispensed, any advice given to the client and any other details on a previously designed data collection form. These forms were filled within 15 min after leaving the pharmacy to enhance the accuracy of data collection. Data were subsequently analyzed using SPSS version 21 and descriptive statistics was used to describe the data.

## Result

Simulated clients visited 353 pharmacies and medical stores out of which 267 were pharmacies and 86 were medical stores. 82.7% pharmacies and medical stores dispensed an antibiotic without demanding any prescription and without investigating the reason for buying the antibiotic (DPR level 1). 14.2% dispensed the requested antibiotic after investigating the disease and condition of the patient (DPR level 2). Overall, 96.9% of all pharmacies and medical stores visited dispensed an antibiotic without a prescription and only 3.1% refused to dispense antibiotics although our simulated clients used all three demands level (Fig. 1). 100% of the refusals came from pharmacies and no refusal came from any medical stores. All the medical stores dispensed the demanded antibiotics at DRP Level 1. However, 77.2% of the pharmacies dispensed the antibiotics at DRP level 1, 18.7% of the pharmacies dispensed the antibiotics at DRP level 2 and 4.1% of all the pharmacies visited denied dispensing any antibiotic despite repeated requests.

From the access group, amoxicillin was most commonly requested antibiotic in 39 visits. In the watch group, ciprofloxacin was demanded in 46 visits. Interestingly, the reserve group antibiotic linezolid was also requested without prescription in 41 cases. Other antibiotics offered are presented in Table 1. The most commonly dispensed dosage form was the tablet. The majority of the pharmacy staff did not inquire about associated symptoms and other important information, and dispensed antibiotics without any inspection. In only 89 visits (25.2%), the attended pharmacy or medical store staff guided patients on how to take their medicine and other information, and in only 11.0% of occasions where the simulated clients asked about any medication history (Table 1).

**Table 1:** Data collected from pharmacy/medical store attendants by Simulated Clients

Questions	Frequency	Percent
**Total Simulated Client Cases**	353	100
**Type of Outlet**		
Medical Store	86	24.4
Pharmacy	267	75.6
**Direct Product Request (DPR) Levels**		
DPR Level 1	292	82.7
PR Level 2	50	14.2
Denied to dispense	11	3.1
**WHO Group**		
ccess Group	112	31.7
Watch Group	174	49.3
Reserve Group	67	19.0
**Antibiotic**		
***Access Group***		
Co-amoxiclav	35	9.9
Amoxicillin	39	8.8
Metronidazole	38	10.8
***Watch Group***		
Ciprofloxacin	46	13.0
Azithromycin	31	8.8
Clarithromycin	21	5.9
Levofloxacin	29	8.2
Cefixime	21	5.9
Ceftriaxone	26	7.4
***Reserve Group***		
Linezolid	41	11.6
Fosfomycin	26	7.4
**Dosage Form**		
ablets	171	82.2
Capsules	60	17.0
Injection	24	6.8
Syrup	4	1.1
**The advice given by staff on how to take medicine**	89	25.2
**Asked about other medicine history**	39	11.0

## Discussion

To the best of our knowledge, this is the first study from Pakistan to quantify the extent of non-prescription sales of antibiotics by using a simulated client technique. As mentioned, the simulated client technique is a well-accepted methodology closer to the real-life scenario as compared with other methods which may lead to deceptive results [56, 62, 63]. Improving the prescribing and dispensing of antibiotics is a decisive step in the global strategy to reduce AMR [64, 65]. Consequently, the development of antibiotic usage guidance should be the prioritized within countries in order to achieve access to safe, affordable and effective antibiotics [15, 66, 67]. To assist with this, the WHO in March 2017 reviewed and updated the 19th WHO Model List of Essential Medicines (EML) and classified antibiotics into 3 different categories called the AwaRe group [18, 19]. However, our current study shows that the dispensing and sale of antibiotics was both illegal and against concerns raised by the AwaRe group. This is similar to other studies where antibiotics are frequently dispensed in pharmacies and medical stores without prescriptions, predominantly for the treatment of supposed bacterial infections [29, 31, 37, 62]. A simple observational study was conducted in 5 sample pharmacies of Islamabad & Rawalpindi in which almost 35% of antibiotics were dispensed without official medical prescription or on pharmacist recommendation [63]. A number of studies have been conducted in other parts of the world describing the rate of dispensing of antibiotic [26, 62, 68, 69]. In a systematic review of 35 community surveys from 5 continents, over-the-counter sale of antibiotics prevailed internationally and accounted for 19–100% outside of northern Europe and North America. Similar to our outcomes, Morgan et al. observed that non-prescription dispensing was common for supposed bacterial and non-bacterial infections. In 2 out of 35 surveys, more than 80% pharmacy staff did not question about drug allergies [25].

Out of the total number of antibiotics demanded, ciprofloxacin, linezolid, amoxicillin, metronidazole and co-amoxiclav were the most dispensed. A study conducted in Riyadh, KSA saw higher sales of amoxicillin/clavulanate [70]. Whilst lower use of amoxicillin/clavulanate is encouraging, there were greater sales of ciprofloxacin in our study which is a concern. There were also concerns that only 11% of patients were asked about other concomitant medicines patients were taking although 25.2% gave advice regarding the taking of their antibiotics. The inability of pharmacy staff to investigate patients about concomitant medicines when dispensing without a prescription poses a considerable risk to patients [71]. As mentioned, dispensing of antimicrobials without a prescription is a key potential cause of antibiotic resistance and irrational antibiotic consumption [72]. There is a lack of effective governmental monitoring of antibiotic prescribing and sale of antibiotics in a number of countries, which is why we are facing growing AMR rates= [63].

The current status of poor antibiotic dispensing in Pakistan requires that pharmacists must now play a key role in ensuring the rational use of antibiotics [73] to reduce future AMR rates, building on the national action plan [17]. Pharmacists must be vigilant in their dispensing and provide all the necessary guidelines regarding drug administration and the spread of AMR in the community, building on initiatives from FIP and the WHO [56, 62, 74, 75]. Pharmacists should feel a responsibility to facilitate the patient in addressing their current illness, often being the most proficient healthcare provider available to patients initially especially in LMICs [69].

There is a concern in countries such as Pakistan where patients have limited income alongside high levels of co-payment and ease of access to medicines in community pharmacies. In view of this, self-medication from pharmacies is the preferred option avoiding physicians’ or consultants’ fees [41, 43, 63], which is similar to many other countries [26, 68]. Consequently, there is a need for multifaceted approaches to addressing this. Firstly, there is a need to educate patients that most infections seen in ambulatory care such as upper respiratory tract infections are viral in origin and will typically resolve without an antibiotic [76–80]. This can be via general awareness and other campaigns [54, 81–83]. Secondly, there is a need to educate pharmacists and their assistants about the appropriate management of infections typically seen in ambulatory care given concerns with their knowledge in a number of countries including Pakistan [32, 50, 53, 55, 84, 85]. Studies have shown that educated pharmacists tend to dispense fewer antibiotics for suspected viral infections such as upper respiratory tract infections [56, 86, 87]. Where necessary, patients should be encouraged to visit physicians for their infection. We have seen the successful implementation of strategies to ban the self-purchasing of antibiotics among countries, although overall their impact has been variable [88]. Overall, multifaceted approaches appear to be most successful including substantial fines and other measures to reduce self-purchasing [88]. However, there are concerns with a total ban on self-purchasing of antibiotics in Pakistan for a number of reasons. Firstly, often patients in Pakistan cannot afford to see both a physician and purchase their medicines - so often they go straight to a pharmacists/ medicine outlet. This is especially the case if they have to give up work to see a physician - with pharmacists staying open later. Secondly, in a few localities especially rural areas, the pharmacies or medical stores may often be the only health care facilities available. Lastly, there is limited number of enforcers for any regulation currently in Pakistan. Consequently, it may be more productive to seek ways to educate the pharmacists/ dispensers and the public in the first place with only seeking to potentially ban self-purchasing of antibiotics that are on the watch and restricted list. Educated pharmacists have worked reasonably well in other LMICs to reduce self-purchasing of antibiotics including the Republic of Srpska and Kenya, with guidelines produced in Srpska [37].

### Limitation

We are aware of a number of limitations with our study. Firstly, we did not distinguish whether the person who dealt with the patient was a pharmacist or another pharmacy worker since both pharmacists and other employees work in community pharmacies. This was because our goal was neither to determine the possible differences between the two nor to investigate the presence of pharmacists. The volunteers didn’t want to appear dubious because, as a result of this, the aim of our study could be compromised. Secondly, our study was directed mostly to pharmacies in a few cities of Pakistan with comparatively better demographic aspects and socioeconomic. Consequently, our findings may not necessarily be generalized to the entire country. Despite this, we believe our findings are robust providing direction for the future.

## Conclusion

The results of this study reveal that the non-prescription sale of antibiotics is still common practice in Pakistan despite the legislation. In majority of the cases, it was possible to acquire a watch group antibiotic without an official prescription. Moreover, the pharmacy staff typically neither questioned the patient about their history nor encouraged the patient to seek medical advice. The current status of antibiotics sale and dispensing practices in local community setups is a complex issue. A multi-dimensional approach is needed to warrant the appropriate sale of antibiotics in the country.

## Supplementary information

**Additional file 1.**

## References

[1] de Kraker ME, Stewardson AJ, Harbarth S (2016). Will 10 million people die a year due to antimicrobial resistance by 2050?. PLoS Med.

[2] Prestinaci F, Pezzotti P, Pantosti A (2015). Antimicrobial resistance: a global multifaceted phenomenon. Pathog Glob Health.

[3] Gandra S, Barter DM, Laxminarayan R (2014). Economic burden of antibiotic resistance: how much do we really know?. Clin Microbiol Infect.

[4] Laxminarayan R, Duse A, Wattal C, Zaidi AK, Wertheim HF, Sumpradit N (2013). Antibiotic resistance—the need for global solutions. Lancet Infect Dis.

[5] Bell BG, Schellevis F, Stobberingh E, Goossens H, Pringle M (2014). A systematic review and meta-analysis of the effects of antibiotic consumption on antibiotic resistance. BMC Infect Dis.

[6] Founou RC, Founou LL, Essack SY (2017). Clinical and economic impact of antibiotic resistance in developing countries: a systematic review and meta-analysis. PLoS One.

[7] Ayukekbong JA, Ntemgwa M, Atabe AN (2017). The threat of antimicrobial resistance in developing countries: causes and control strategies. Antimicrob Resist Infect Control.

[8] O’Neill J (2015). Securing new drugs for future generations: the pipeline of antibiotics. The review of antimicrobial resistance.

[9] Freire-Moran L, Aronsson B, Manz C, Gyssens IC, So AD, Monnet DL (2011). Critical shortage of new antibiotics in development against multidrug-resistant bacteria—time to react is now. Drug Resist Updat.

[10] Van Boeckel TP, Gandra S, Ashok A, Caudron Q, Grenfell BT, Levin SA (2014). Global antibiotic consumption 2000 to 2010: an analysis of national pharmaceutical sales data. Lancet Infect Dis.

[11] Ventola CL (2015). The antibiotic resistance crisis: part 1: causes and threats. Pharm Ther.

[12] Laxminarayan R, Sridhar D, Blaser M, Wang M, Woolhouse M (2016). Achieving global targets for antimicrobial resistance. Science..

[13] World Health Organization (2015). World health assembly addresses antimicrobial resistance, immunization gaps and malnutrition.

[14] Shallcross LJ, Howard SJ, Fowler T, Davies SC (2015). Tackling the threat of antimicrobial resistance: from policy to sustainable action. Philos Trans R Soc B.

[15] Jinks T, Lee N, Sharland M, Rex J, Gertler N, Diver M (2016). A time for action: antimicrobial resistance needs global response. Bull World Health Organ.

[16] Seale AC, Gordon NC, Islam J, Peacock SJ, Scott JAG (2017). AMR surveillance in low and middle-income settings-a roadmap for participation in the global antimicrobial surveillance system (GLASS). Wellcome Open Res.

[17] Saleem Z, Hassali MA, Hashmi FK (2018). Pakistan’s national action plan for antimicrobial resistance: translating ideas into reality. Lancet Infect Dis.

[18] World Health Organization. The selection and use of essential medicines: report of the WHO Expert Committee, 2017 (including the 20th WHO Model List of Essential Medicines and the 6th Model List of Essential Medicines for Children): World Health Organization; 2017.

[19] Sharland M, Pulcini C, Harbarth S, Zeng M, Gandra S, Mathur S (2018). Classifying antibiotics in the WHO essential medicines list for optimal use—be AWaRe. Lancet Infect Dis.

[20] Le Doare K, Barker CI, Irwin A, Sharland M (2015). Improving antibiotic prescribing for children in the resource-poor setting. Br J Clin Pharmacol.

[21] Shaikhan F, Rawaf S, Majeed A, Hassounah S (2018). Knowledge, attitude, perception and practice regarding antimicrobial use in upper respiratory tract infections in Qatar: a systematic review. JRSM open.

[22] Sakeena M, Bennett AA, McLachlan AJ (2018). Non-prescription sales of antimicrobial agents at community pharmacies in developing countries: a systematic review. Int J Antimicrob Agents.

[23] Hoa NQ, Chuc NTK, Phuc HD, Larsson M, Eriksson B, Lundborg CS (2011). Unnecessary antibiotic use for mild acute respiratory infections during 28-day follow-up of 823 children under five in rural Vietnam. Trans R Soc Trop Med Hyg.

[24] Md Rezal RS, Hassali MA, Alrasheedy AA, Saleem F, Md Yusof FA, Godman B (2015). Physicians’ knowledge, perceptions and behaviour towards antibiotic prescribing: a systematic review of the literature. Expert Rev Anti-Infect Ther.

[25] Morgan DJ, Okeke IN, Laxminarayan R, Perencevich EN, Weisenberg S (2011). Non-prescription antimicrobial use worldwide: a systematic review. Lancet Infect Dis.

[26] Al-Faham Z, Habboub G, Takriti F (2011). The sale of antibiotics without prescription in pharmacies in Damascus, Syria. J Infect Dev Ctries.

[27] Safrany N, Monnet DL (2012). Antibiotics obtained without a prescription in Europe. Lancet Infect Dis.

[28] Saleem Z, Saeed H, Ahmad M, Yousaf M, Hassan HB, Javed A (2016). Antibiotic self-prescribing trends, experiences and attitudes in upper respiratory tract infection among pharmacy and non-pharmacy students: a study from Lahore. PLoS One.

[29] Auta A, Hadi MA, Oga E, Adewuyi EO, Abdu-Aguye SN, Adeloye D (2019). Global access to antibiotics without prescription in community pharmacies: a systematic review and meta-analysis. J Inf Secur.

[30] Ocan M, Obuku EA, Bwanga F, Akena D, Richard S, Ogwal-Okeng J (2015). Household antimicrobial self-medication: a systematic review and meta-analysis of the burden, risk factors and outcomes in developing countries. BMC Public Health.

[31] Nepal G, Bhatta S (2018). Self-medication with antibiotics in WHO southeast Asian region: a systematic review. Cureus.

[32] Servia-Dopazo M, Figueiras A (2018). Determinants of antibiotic dispensing without prescription: a systematic review. J Antimicrob Chemother.

[33] Napolitano F, Izzo MT, Di Giuseppe G, Angelillo IF (2013). Public knowledge, attitudes, and experience regarding the use of antibiotics in Italy. PLoS One.

[34] Oh AL, Hassali MA, Al-Haddad MS, Sulaiman SAS, Shafie AA, Awaisu A (2010). Public knowledge and attitudes towards antibiotic usage: a cross-sectional study among the general public in the state of Penang, Malaysia. J Infect Dev Ctries.

[35] World Health Organization (2015). Antibiotic resistance: multi-country public awareness survey.

[36] Bahnassi A (2015). A qualitative analysis of pharmacists’ attitudes and practices regarding the sale of antibiotics without prescription in Syria. J Taibah Univ Med Sci.

[37] Torres N, Chibi B, Middleton L, Solomon V, Mashamba-Thompson T (2019). Evidence of factors influencing self-medication with antibiotics in low and middle-income countries: a systematic scoping review. Public Health.

[38] Kalungia A, Godman B (2019). Nonprescription antibiotic sales in China and the implications. Lancet Infect Dis.

[39] Ramay BM, Lambour P, Cerón A (2015). Comparing antibiotic self-medication in two socio-economic groups in Guatemala City: a descriptive cross-sectional study. BMC Pharmacol Toxicol.

[40] Miller R, Goodman C (2016). Performance of retail pharmacies in low- and middle-income Asian settings: a systematic review. Health Policy Plan.

[41] Bilal M, Haseeb A, Khan MH, Arshad MH, Ladak AA, Niazi SK (2016). Self-medication with antibiotics among people dwelling in rural areas of Sindh. J Clin Diagn Res.

[42] Riaz H, Godman B, Hussain S, Malik F, Mahmood S, Shami A (2015). Prescribing of bisphosphonates and antibiotics in Pakistan: challenges and opportunities for the future. JPHSR.

[43] Aziz MM, Masood I, Yousaf M, Saleem H, Ye D, Fang Y (2018). Pattern of medication selling and self-medication practices: a study from Punjab, Pakistan. PLoS One.

[44] Ali IKA (2015). Self-medication of antibiotics: a perspective on alarming situation in Peshawar, Khyber Pakhtunkhwa, Pakistan. Arch Pharm Pract.

[45] Javed MP (2013). Self-medication of anti-biotics amongst university students of Islamabad: prevalence, knowledge and attitudes.

[46] Shah SJ, Ahmad H, Rehan RB, Najeeb S, Mumtaz M, Jilani MH (2014). Self-medication with antibiotics among non-medical university students of Karachi: a cross-sectional study. BMC Pharmacol Toxicol.

[47] Hussain A, Ibrahim MI, Baber ZD (2012). Compliance with legal requirements at community pharmacies: a cross sectional study from Pakistan. Int J Pharm Pract.

[48] Gillani A, Ji W, Hussain W, Imran A, Chang J, Yang C (2017). Antibiotic self-medication among non-Medical University students in Punjab, Pakistan: a Cross-sectional survey. Int J Environ Res Public Health.

[49] Khan SA (2015). Manual of drug Laws.

[50] Sarwar MR, Saqib A, Iftikhar S, Sadiq T (2018). Knowledge of community pharmacists about antibiotics, and their perceptions and practices regarding antimicrobial stewardship: a cross-sectional study in Punjab, Pakistan. Infect Drug Resist.

[51] Atif M, Asghar S, Mushtaq I, Malik I, Amin A, Babar Z-U-D (2019). What drives inappropriate use of antibiotics? A mixed methods study from Bahawalpur, Pakistan. Infect Drug Resist.

[52] Shaikh BT (2017). Anti-microbial resistance in Pakistan: a public health issue. J Ayub Med Coll Abbottabad.

[53] Waseem H, Ali J, Sarwar F, Khan A, Rehman HSU, Choudri M (2019). Assessment of knowledge and attitude trends towards antimicrobial resistance (AMR) among the community members, pharmacists/pharmacy owners and physicians in district Sialkot, Pakistan. Antimicrob Resist Infect Control.

[54] Elong Ekambi G-A, Okalla Ebongue C, Penda IC, Nnanga Nga E, Mpondo Mpondo E, Eboumbou Moukoko CE (2019). Knowledge, practices and attitudes on antibiotics use in Cameroon: self-medication and prescription survey among children, adolescents and adults in private pharmacies. PLoS One.

[55] Zawahir S, Lekamwasam S, Aslani P (2019). A cross-sectional national survey of community pharmacy staff: knowledge and antibiotic provision. PLoS One.

[56] Markovic-Pekovic V, Grubisa N, Burger J, Bojanic L, Godman B (2017). Initiatives to reduce nonprescription sales and dispensing of antibiotics: findings and implications. J Res Pharm Pract.

[57] Weiss MC, Booth A, Jones B, Ramjeet S, Wong E (2010). Use of simulated patients to assess the clinical and communication skills of community pharmacists. Pharm World Sci.

[58] Berger K, Eickhoff C, Schulz M (2005). Counselling quality in community pharmacies: implementation of the pseudo customer methodology in Germany. J Clin Pharm Ther.

[59] Alabid AHM, Ibrahim MIM, Hassali MA (2014). Antibiotics dispensing for URTIs by community pharmacists (CPs) and general medical practitioners in Penang, Malaysia: a comparative study using simulated patients (SPs). J Clin Diagn Res.

[60] Ibrahim MI, Palaian S, Al-Sulaiti F, El-Shami S (2016). Evaluating community pharmacy practice in Qatar using simulated patient method: acute gastroenteritis management. Pharm Pract.

[61] Chang J, Xu S, Zhu S, Li Z, Yu J, Zhang Y, et al. Using simulated clients to assess nonprescription antibiotic dispensing at community pharmacies in China: a mixed Cross-sectional and longitudinal study. Lancet Infect Dis. 2019; In Press. 10.1016/S1473-3099(19)30324-X 31588042

[62] Llor C, Cots JM (2009). The sale of antibiotics without prescription in pharmacies in Catalonia, Spain. Clin Infect Dis.

[63] Ashraf F, Hafeez A, Imtiaz F, Ayub A, Imtiaz H (2017). Antibiotic dispensing and prescription pattern in pharmacies of Islamabad and Rawalpindi: Pakistan. Int J Collab Res Intern Med Public Health.

[64] Mendelson M, Matsoso MP (2015). The World Health Organization global action plan for antimicrobial resistance. SAMJ.

[65] World Health Organisation (2018). Antimicrobial resistance.

[66] Lee BX, Kjaerulf F, Turner S, Cohen L, Donnelly PD, Muggah R (2016). Transforming our world: implementing the 2030 agenda through sustainable development goal indicators. J Public Health Policy.

[67] Essack SY, Desta AT, Abotsi RE, Agoba EE (2017). Antimicrobial resistance in the WHO African region: current status and roadmap for action. J Public Health (Oxford, England).

[68] Chang J, Ye D, Lv B, Jiang M, Zhu S, Yan K (2017). Sale of antibiotics without a prescription at community pharmacies in urban China: a multicentre cross-sectional survey. J Antimicrob Chemother.

[69] Nyazema N, Viberg N, Khoza S, Vyas S, Kumaranayake L, Tomson G (2007). Low sale of antibiotics without prescription: a cross-sectional study in Zimbabwean private pharmacies. J Antimicrob Chemother.

[70] Bin Abdulhak AA, Altannir MA, Almansor MA, Almohaya MS, Onazi AS, Marei MA (2011). Non prescribed sale of antibiotics in Riyadh, Saudi Arabia: a cross sectional study. BMC Public Health.

[71] Khurshid HF, Zikria S, Hamid S, Kumar VA (2016). Self-medication of corticosteroids: a life threatening case report from Pakistan. J Pharm Pract Commun Med.

[72] Plachouras D, Kavatha D, Antoniadou A, Giannitsioti E, Poulakou G, Kanellakopoulou K (2010). Dispensing of antibiotics without prescription in Greece, 2008: another link in the antibiotic resistance chain. Eurosurveillance..

[73] Hadi MA, Karami NA, Al-Muwalid AS, Al-Otabi A, Al-Subahi E, Bamomen A (2016). Community pharmacists’ knowledge, attitude, and practices towards dispensing antibiotics without prescription (DAwP): a cross-sectional survey in Makkah Province, Saudi Arabia. Int J Infect Dis.

[74] WHO. The role of pharmacist in encouraging prudent use of antibiotics and averting antimicrobial resistance: a review of policy and experience. Available at URL: http://www.euro.who.int/__data/assets/pdf_file/0006/262815/The-role-of-pharmacist-in-encouraging-prudent-use-of-antibiotics-and-averting-antimicrobial-resistance-a-review-of-policy-and-experience-Eng.pdf?ua=1.

[75] FIP. FIP statement of policy - control of antimicrobial medicines resistance (AMR). Available from URL: http://www.fip.org/www/uploads/database_file.php?id=289&table_id.

[76] Dyar OJ, Beovic B, Vlahovic-Palcevski V, Verheij T, Pulcini C (2016). How can we improve antibiotic prescribing in primary care?. Expert Rev Anti-Infect Ther.

[77] Havers FP, Hicks LA, Chung JR, Gaglani M, Murthy K, Zimmerman RK (2018). Outpatient antibiotic prescribing for acute respiratory infections during influenza seasons. JAMA Netw Open.

[78] Barnett ML, Linder JA (2014). Antibiotic prescribing for adults with acute bronchitis in the United States, 1996–2010. Jama..

[79] Ebell MH, Radke T (2015). Antibiotic use for viral acute respiratory tract infections remains common. Am J Manag Care.

[80] Butler CC, Hood K, Verheij T, Little P, Melbye H, Nuttall J (2009). Variation in antibiotic prescribing and its impact on recovery in patients with acute cough in primary care: prospective study in 13 countries. BMJ (Clinical research ed).

[81] Cross EL, Tolfree R, Kipping R (2017). Systematic review of public-targeted communication interventions to improve antibiotic use. J Antimicrob Chemother.

[82] Huttner B, Goossens H, Verheij T, Harbarth S (2010). Characteristics and outcomes of public campaigns aimed at improving the use of antibiotics in outpatients in high-income countries. Lancet Infect Dis.

[83] WHO Collaborating Centre on Patient Safety (2017). The University of Geneva hospitals and Faculty of Medicine. Evaluation of antibiotic awareness campaigns.

[84] Hoxha I, Malaj A, Kraja B, Bino S, Oluka M, Markovic-Pekovic V (2018). Are pharmacists’ good knowledge and awareness on antibiotics taken for granted? The situation in Albania and future implications across countries. J Glob Antimicrob Resist.

[85] Ahmad A, Khan MU, Moorthy J, Jamshed SQ, Patel I (2015). Comparison of knowledge and attitudes about antibiotics and resistance, and antibiotics self-practicing between bachelor of pharmacy and doctor of pharmacy students in southern India. Pharm Pract.

[86] Mukokinya M, Opanga S, Oluka M, Godman B (2018). Dispensing of antimicrobials in Kenya: a cross-sectional pilot study and its implications. J Res Pharm Pract.

[87] Eslami N, Eshraghi A, Vaseghi G, Mehdizadeh M, Masjedi M, Mehrpooya M (2016). Pharmacists’ knowledge and attitudes towards upper respiratory infections (URI) in Iran: a Cross sectional study. Rev Recent Clin Trials.

[88] Jacobs TG, Robertson J, van den Ham HA, Iwamoto K, Bak Pedersen H, Mantel-Teeuwisse AK (2019). Assessing the impact of law enforcement to reduce over-the-counter (OTC) sales of antibiotics in low- and middle-income countries; a systematic literature review. BMC Health Serv Res.

