# Supplementary material for: Sale of WHO AWaRe groups antibiotics without a prescription in Pakistan: a simulated client study
Source: J Pharm Policy Pract. 2020 Aug 3;13:26. doi: 10.1186/s40545-020-00233-3 (PMC7397594; doi:10.1186/s40545-020-00233-3)
Supplement: Supplementary file 1 — Additional file 1. [file 40545_2020_233_MOESM1_ESM.docx]

**ANTIBIOTIC SALE WITHOUT PRESCRIPTION**

**Important notes for students:**

- Visit pharmacy/medical store of your own home town.
- Read questionnaire thoroughly before visiting pharmacy/medical store and do not show the questionnaire to pharmacy/medical store staff.
- Memorize the answers of all questions.
- Fill the form properly within 15 minutes after you leave the pharmacy/medical store.
- Do not visiting hospital pharmacies.

**PART 1**

**Questions related to pharmacy/ medical store**

- **City:**
- **Select one**

1. Pharmacy b. Medical store

- **Type of pharmacy/ medical store**

a. Individual b. Chain

- **Pharmacist availability**

a. Yes b. No

- **Gender (pharmacist)**

a. Male b. Female

- **Select the case scenario (Part 2)**

1. 1 b. 2

**PART 2**

**CASE SCENARIO 1 (RESPIRATORY TRACT INFECTION)**

**DEMAND LEVEL 1**

SC (simulated client) will show that he/she is a brother (……) of 23 years (….. years) old boy who has upper respiratory tract infection from 3 days. SC will say that he has fever, runny nose and cough; could you give me something for treatment?

- Medicine received:

1. Yes b. No

If yes, then fill PART 3

If no, then go to next level:

**DEMAND LEVEL 2**

Answer to the questions which may asked by pharmacy staff

**SYMPTOMS OF DISEASE**: Sore throat, head ache, tiredness and weakness

**HE TAKES SOME MEDICINE OR ANYTHING**: No medicine, only using ginger qahwa

**DRUG ALLERGY HISTORY**: No

**MEDICAL HISTORY**: No

**HE VISITED DOCTOR**: No

**WHY**: Didn’t have too much time/ feeling weak, can’t go to doctor or wait for my appointment.

**HAVE PRESCRIPTION**: no

It’s just a minor illness. I think if you give me some antibiotics it will work and then there will be no need to see doctor, kindly give me some antibiotic to alleviate the symptoms

- Antibiotic received:

1. yes b. no

If yes, then fill PART 3

If no, then go to next level

**DEMAND LEVEL3**

Give me erythromycin or ampicillin

- Antibiotic received:

1. yes b. no

If yes, then fill PART 3

If no, then ask about reason of refusal.

**REASON OF REFUSION**

a. condition doesn’t require any drug

b. require prescription

c. refer to some doctor

**CASE SCENARIO 2 (DIARRHEA)**

**DEMAND LEVEL 1**

Student acting as the neighbor of 26 years old man comes to medical store/pharmacy asking medicines for the person who is having diarrhea. The person enters the pharmacy and goes as: “Hello, my neighbor is having loose stools since last night, can you get me any medicine to relieve the symptoms?

- Antibiotic received:

1. yes b. no

If yes, then fill PART 3

If no, then go to next level

**DEMAND LEVEL 2**

**SYMPTOMS:** Person is feeling weak, he’s having diarrhea since last night and also has slight fever, but he doesn’t have headache, vomiting, nausea or any blood in stools.

**HE TAKES SOME MEDICINE OR ANYTHING**: No medicine, only using ORS.

**DRUG ALLERGY HISTORY**: No

**MEDICAL HISTORY**: No

**VISITED ANY DOCTOR**: No

**WHY**: Didn’t have too much time/ feeling weakness, can’t go to doctor or wait for my appointment.

**HAVE PRESCRIPTION**: No

It’s just a minor illness. I think if you give me some antibiotics it will work and then there will be no need to see doctor, kindly give me some antibiotic to alleviate the symptoms.

- Antibiotic received:

1. yes b. no

If yes, then fill PART 3

If no, then go to next level

**DEMAND LEVEL 3**

Give me loperamide or ciprofloxacin

- Antibiotic received:

1. yes b. no

If yes, then fill PART 3

If no, then ask about reason of refusal

**REASON OF REFUSION**

a. condition doesn’t require any drug

b. require prescription

c. refer to some doctor

**PART 3**

**Questions related to drug received:**

- Antibiotic Included: a. Yes b. No
- If “No” then finish the survey after writing brands
- if “Yes” then answer following questions
- Antibiotic brand name:
- Generic:
- Dosage form:
- Strength:
- Advice given by pharmacist/salesman on how to take medicine

1. Yes b. No

- Asked any other question

a. Yes b. No

**Imp Points:**

- If someone at your home does need any antibiotic and has a prescription, then simply go to medical store and ask for antibiotic without showing prescription.
- You can pay through your pocket money
- Purchase not more than three tablets/capsules.
- Make some excuse

**AWaRe Group Classification**

**1. Access Group Antibiotics**

| **6.2.1** **Beta-lactam** **medicines** | | **6.2.2** **Other** **antibacterials** | |
| --- | --- | --- | --- |
| amoxicillin | cefotaxime* | amikacin | gentamicin |
| amoxicillin + clavulanic acid | ceftriaxone* | azithromycin* | metronidazole |
| ampicillin | cloxacillin | chloramphenicol | nitrofurantoin |
| benzathine benzylpenicillin | phenoxymethylpenicillin | ciprofloxacin* | spectinomycin (EML only) |
| benzylpenicillin | piperacillin + tazobactam* | clarithromycin* | sulfamethoxazole + trimethoprim |
| cefalexin | procaine benzyl penicillin | clindamycin | vancomycin (oral)* |
| cefazolin | *meropenem** | doxycycline | *vancomycin* *(parenteral)** |
| cefixime* |  |  |  |

*Italics* *=* *complementary* *list*

*Watch group antibiotics included in the EML/EMLc only for specific, limited indications

**2. Watch Group Antibiotics**

| **Watch** **group** **antibiotics** |
| --- |
| Quinolones and fluoroquinolones  e.g. ciprofloxacin, levofloxacin, moxifloxacin, norfloxacin |
| 3rd-generation cephalosporins (with or without beta-lactamase inhibitor) e.g. cefixime, ceftriaxone, cefotaxime, ceftazidime |
| Macrolides  e.g. azithromycin, clarithromycin, erythromycin |
| Glycopeptides  e.g. teicoplanin, vancomycin |
| Antipseudomonal penicillins + beta-lactamase inhibitor e.g. piperacillin-tazobactam |
| Carbapenems  e.g. meropenem, imipenem + cilastatin |
| Penems  e.g. faropenem |

**3. Reserve Group Antibiotics**

| **Reserve** **group** **antibiotics** |
| --- |
| Aztreonam |
| 4th generation cephalosporins e.g. cefepime |
| 5th generation cephalosporins e.g. ceftaroline |
| Polymyxins e.g. polymyxin B, colistin |
| Fosfomycin (IV) |
| Oxazolidinones e.g. linezolid |
| Tigecycline  Daptomycin |
